# Refractory Abdominal Pain in a Patient with Chronic Lymphocytic Leukemia: Be Wary of Acquired Angioedema due to C1 Esterase Inhibitor Deficiency

**DOI:** 10.1155/2018/7809535

**Published:** 2018-01-10

**Authors:** Abdullateef Abdulkareem, Ryan S. D'Souza, Joshua Mundorff, Pragya Shrestha, Oluwaseun Shogbesan, Anthony Donato

**Affiliations:** ^1^Department of Medicine, Reading Hospital, West Reading, PA, USA; ^2^Department of Anesthesiology, Mayo Clinic Hospital, Rochester, MN, USA

## Abstract

Acquired angioedema due to C1 inhibitor deficiency (C1INH-AAE) is a rare and potentially fatal syndrome of bradykinin-mediated angioedema characterized by episodes of angioedema without urticaria. It typically manifests with nonpitting edema of the skin and edema in the gastrointestinal (GI) tract mucosa or upper airway. Edema of the upper airway and tongue may lead to life-threatening asphyxiation. C1INH-AAE is typically under-diagnosed because of its rarity and its propensity to mimic more common abdominal conditions and allergic reactions. In this article, we present the case of a 62-year-old male with a history of recently diagnosed chronic lymphocytic leukemia (CLL) who presented to our hospital with recurrent abdominal pain, initially suspected to have *Clostridium difficile* colitis and diverticulitis. He received a final diagnosis of acquired angioedema due to C1 esterase inhibitor deficiency due to concomitant symptoms of lip swelling, cutaneous nonpitting edema of his lower extremities, and complement level deficiencies. He received acute treatment with C1 esterase replacement and icatibant and was maintained on C1 esterase infusions. He also underwent chemotherapy for his underlying CLL and did not experience further recurrence of his angioedema.

## 1. Introduction

Acquired angioedema due to C1 esterase inhibitor deficiency (C1INH-AAE) is a rare and potentially fatal disorder caused by acquired consumption of C1 esterase inhibitor. Studies estimate a prevalence rate between 1 in 100,000 and 1 in 500,000 patients, although it may be higher as the condition is commonly unrecognized [1]. It may manifest with nonpitting edema of the skin, including the skin of the face, lips, limbs, or genitals, abdominal pain secondary to edema of the gastrointestinal mucosa, and severe life-threatening edema of the upper airway and oral mucosa [2].

## 2. Case Presentation

A 62-year-old male with a two-month history of recurrent hospital admissions for abdominal pain presented to the emergency room with colicky, generalized abdominal pain. He also reported new asymmetric swelling of his upper and lower lips. He denied associated difficulty swallowing or breathing. He had no previous history of angioedema, food allergies, or new medications. He denied any fever, chills, malaise, trauma, nausea, vomiting, diarrhea, constipation, hematochezia or melena, weight loss, sick contacts, neurological deficits, or recent travel history. Of note, he had never taken angiotensin-converting enzyme inhibitors (ACEi) or angiotensin receptor blocker medications. He was a former smoker and quit 14 years ago and reported only occasional alcohol intake. He had no family history of angioedema. This was the third hospital admission for abdominal pain episodes within six weeks, with prior episodes spontaneously resolving within a few days.

Six weeks prior to current presentation, a computed tomography (CT) scan of the abdomen for a prior bout of this illness revealed diffuse small bowel thickening with mild adjacent mesenteric fat stranding and a thickened, nodular terminal ileum and cecum. He also had a positive *Clostridium difficile* stool toxin test. He was treated with metronidazole for *C. difficile* colitis although diarrhea was not a prominent symptom. Two weeks prior to current presentation, he was admitted for recurrent abdominal pain for which another CT was performed, and on this one, he was found to have severe jejunal edema, new proctocolitis, and several enlarged mesenteric and inguinal lymph nodes. The lymph nodes were biopsied and found to have CD5-positive cells consistent with a new diagnosis of chronic lymphocytic leukemia/small lymphocytic lymphoma (CLL/SLL), later confirmed by bone marrow biopsy. The patient was again treated for presumed *C. difficile* colitis, due to another positive stool toxin test, this time with oral vancomycin. He also received a course of amoxicillin-clavulanate as CT abdomen showed extensive diverticulosis and evidence of sigmoid colitis, introducing the possibility of diverticulitis. Evaluation during that admission included esophagogastroduodenoscopy (EGD) and push enteroscopy. Both were unremarkable except for previously known Barrett's esophagus. Interestingly, he also had asymmetric left lower limb cutaneous swelling and pain during that admission that had an unrevealing workup including a negative Doppler ultrasound.

**Figure 1:**
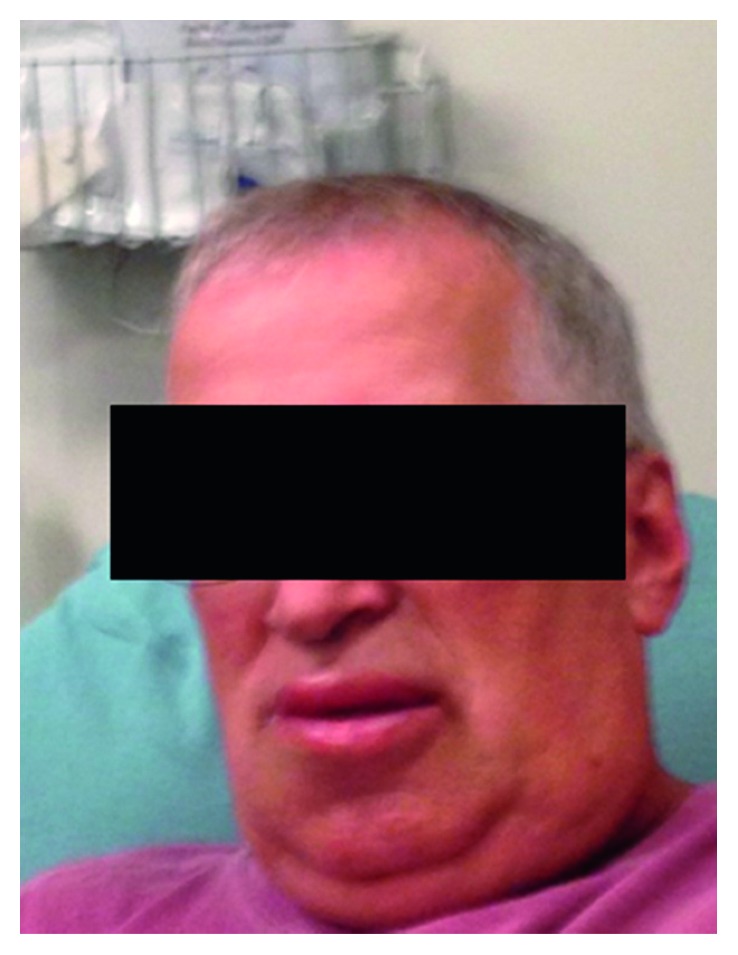
Patient with symmetric lip swelling of upper and lower lips.

During the current presentation, vital signs were normal with oxygen saturation at 95% on room air. Physical examination revealed lungs that were clear to auscultation without stridor. Symmetric upper and lower lip swelling without hives was noted (Figure 1). The central abdomen was mildly tender to palpation two inches above the umbilicus with no guarding or rebound. No edema was noted in the lower extremities during this current admission. All other systems examined were normal.

## 3. Investigations

**Figure 2:**
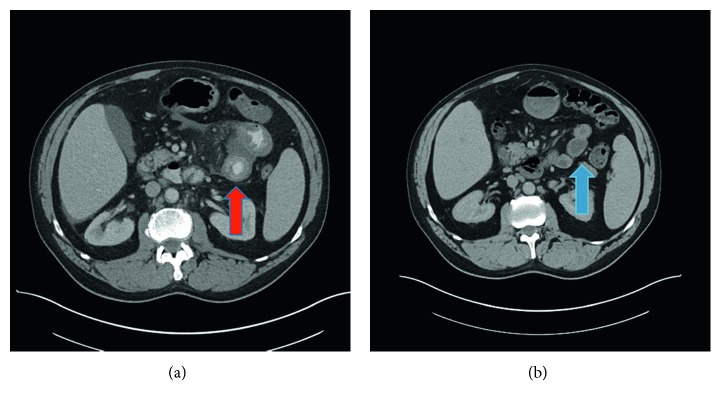
CT abdomen findings before and after treatment. (a) Before treatment, the patient was noted to have jejunal enteritis, which is indicated by the target sign (red arrow). (b) After treatment, the patient had resolution of jejunitis (blue arrow).

Complete blood count revealed a white blood count of 4900/*μ*L (normal: 4,800–10,800/*μ*L), hemoglobin of 12.4 (normal: 14–17.5 g/dL), and platelets of 149,000 (normal: 130,000–400,000/*μ*L). Lactic acid, sedimentation rate, C-reactive protein, lipase, and liver function tests were normal. Considering the bouts of unexplained bowel edema and swelling lips without hives, a diagnostic workup for angioedema was pursued. Functional C1 esterase inhibitor level was found to be 4% (normal: >40%), along with C1 esterase inhibitor antigen of <3 mg/dl (normal: 21–39 mg/dl), C4 complement < 1.7 mg/dl (normal: 12–38 mg/dl), and low normal C1q level of 113 *μ*g/ml (normal: 109–242 *μ*g/ml). C3 complement level was normal at 88.6 mg/dl (normal: 59–152 mg/dl). *C. difficile* stool toxin test during this admission was negative. Stool samples were also negative for *Salmonella*, *Shigella*, *Escherichia coli*, *Campylobacter*, *Yersinia enterocolitica*, *Giardia*, *Vibrio*, and *Aeromonas*. CT abdomen and pelvis (Figure 2) showed jejunal enteritis with progressive duodenitis and stable abdominopelvic lymphadenopathy. A repeat C1q level performed 6 months later was low at <50 *μ*g/ml (109–242 *μ*g/ml).

## 4. Differential Diagnosis

Given the patient's history of positive *C. difficile* stool tests, a diagnosis of *C. difficile* colitis was initially assigned, although repeat stool toxin assay was negative. Diverticulitis, acute pancreatitis, peptic ulcer disease, acute cholecystitis, and inflammatory bowel syndrome were excluded based on clinical history, negative biochemical and stool tests, EGD, and CT abdomen/pelvis results. Stool studies were negative for bacterial or parasitic infection that could otherwise explain the CT scan findings. Viral gastroenteritis was considered although patient denied any nausea, vomiting, or diarrhea, and symptoms appeared to be recurrent.

Since nonspecific abdominal pain was accompanied with symptoms of lip swelling and recent cutaneous swelling in his lower extremities, a suspicion was formed of angioedema. Allergic angioedema was excluded as the patient denied urticaria and exposure to precipitating agents and had abnormal complement levels in his serum. Similarly, the presence of low C1-INH levels [3] and the absence of ACEi use excluded ACEi-induced angioedema. Hereditary angioedema (HAE) was unlikely because our patient denied a family history of angioedema and experienced onset of symptoms in his 6th decade, as opposed to before the 2nd decade which is more typical of HAE [4].

## 5. Treatment

Patient was urgently administered intravenous methylprednisolone 125 mg and benadryl. Once a diagnosis of acquired C1 esterase deficiency was made, he received C1 esterase replacement and icatibant (a bradykinin B_2_ receptor antagonist). He was subsequently maintained on C1 esterase replacement therapy and given rescue injections of icatibant for acute exacerbations. He also received outpatient chemotherapy for his CLL/SLL with six cycles of rituximab, cyclophosphamide, vincristine, and prednisone (R-CVP). He had no further episodes of abdominal pain, lip swelling, or lower extremity swelling. Interval imaging with CT abdomen showed resolution of jejunitis and duodenitis (Figure 2).

## 6. Discussion

Approximately half of patients with C1INH-AAE may have upper airway edema, and consequently death from asphyxiation or anoxic brain injury can occur in up to 30% of patients [3, 5, 6]. However, more benign and nonspecific presentations of GI angioedema and cutaneous edema may be the only manifesting symptoms. GI symptoms of angioedema include nonspecific colicky abdominal pain, vomiting, diarrhea, and abdominal distension [2], which can be mistaken for common abdominal conditions such as gastroenteritis and diverticulitis. Cutaneous edema may manifest as nonpitting edema of the skin, usually affecting the face but may also involve the lower extremities, mimicking allergic reactions and thrombophlebitis.

Because the diagnosis is extremely uncommon and its symptoms are protean, there is frequently a delay in diagnosis. A retrospective nationwide study in France found a median delay in diagnosis of 10 months [3], whereas a national audit in the United Kingdom showed a diagnostic delay of 5 years [7]. Our patient was diagnosed within only 2 months of symptom onset, but after three hospital admissions and incorrect diagnoses of *C. difficile colitis* and diverticulitis.

The key pathologic abnormality in C1INH-AAE is the acquired deficiency of C1-inhibitor enzyme (C1-INH). A major function of C1-INH is the inhibition of kallikrein, which is a protease that cleaves kininogen and releases bradykinin [8]. Consequently, in C1-INH deficiency, the absence of inhibition of kallikrein leads to increased bradykinin levels, which then facilitate vasodilation and increased tissue permeability, resulting in angioedema flares [8]. C1-INH deficiency in acquired angioedema (AAE) may be caused by excessive consumption due to hyperactivation of the classic complement pathways associated with underlying lymphoproliferative diseases or by autoantibodies (autoimmune mediated). Both lymphoproliferative disorders and autoantibodies frequently coexist in the same patients and one may lead to the other [9–11]. Our patient did not have antibodies tested, but he did have CLL (a lymphoproliferative disease).

Laboratory findings include low C1-INH activity, low C3 and C4 levels, and autoantibodies to C1-INH in up to 70% of patients with AAE [12, 13]. C1q level is typically low in up to 70% of patients, although it may be normal in early stages of the disease [14]. Our patient had low C1-INH activity, low C1-INH level, low C4 complement, and an initially low-normal level of C1q but low repeat C1q levels six months after diagnosis.

Most patients afflicted with C1INH-AAE also have concomitant lymphoproliferative and autoimmune disorders [15]. In one review that included 136 cases of acquired C1 inhibitor deficiency, lymphatic malignancies were identified in 35%, MGUS in 32%, autoimmune diseases in 8%, with adenocarcinoma, and other malignancies identified in 6% [15]. Lymphoproliferative disorders and B-cell malignancies are the most commonly associated conditions [10, 16], especially splenic marginal zone lymphomas [17]. Associated autoimmune disorders include systemic lupus erythematosus, cryoglobulinemia, and autoimmune hemolytic anemia [18, 19]. Interestingly, there have also been reports of association with *Helicobacter pylori* [20]. In our case, the patient had been recently diagnosed with CLL, a non-Hodgkin's lymphoma with known association with C1INH-AAE [13, 21–23].

Primary pharmacologic treatment for acute C1INH-AAE includes C1 esterase inhibitor concentrate from human plasma or recombinant C1 inhibitor concentrate, icatibant (a synthetic bradykinin B_2_ receptor antagonist), and/or ecallantide (a kallikrein inhibitor). The most widely used therapy is C1 esterase inhibitor concentrate, although resistance to this treatment over time, necessitating higher doses to control symptoms, has been reported [10].

Importantly, unlike more common causes of angioedema including allergic and drug-induced angioedema, acute presentations of C1INH-AAE typically do not respond to corticosteroids, antihistamine, or epinephrine [24]. Prophylactic treatment to prevent recurrent symptoms of C1INH-AAE includes C1 esterase inhibitor concentrate administered every 3-4 days, attenuated androgens (i.e., danazol and stanozolol) and antifibrinolytic agents [17, 25]. Our index patient received C1 esterase inhibitor concentrate and icatibant twice weekly prophylaxis with C1 esterase inhibitor concentrate. He experienced resolution of his symptoms following initiation of that therapy and treatment of his lymphoma with chemotherapy. Other case reports have documented similar improvement in angioedema symptoms following treatment of lymphoma [26].

Physicians caring for patients with unexplained abdominal pain especially with concomitant skin, lip, or airway swelling should consider angioedema as a possibility and should check laboratory testing for confirmation.

## 7. Teaching Points

1. Wider appreciation of the possibility of C1INH-AAE in refractory recurrent abdominal pain, particularly in patients with lymphoproliferative disorders, could lead to proper and timely medical therapy and avert fatal respiratory complications from laryngeal and upper airway edema.
2. Abdominal symptoms in C1INH-AAE may mimic gastroenteritis and diverticulitis, whereas nonpitting cutaneous edema may be mistaken for allergic reactions and thrombophlebitis.
3. Unlike more common causes of angioedema including allergic and drug-induced angioedema, acute presentations of angioedema secondary to C1 esterase deficiency typically do not respond to corticosteroids, antihistamine, or epinephrine and necessitate treatment with C1 inhibitor replacement therapy, icatibant, ecallantide, or fresh frozen plasma.
4. If an underlying associated disease is identified in a patient with C1INH-AAE, including lymphoproliferative malignancy or autoimmune disease, treatment of the underlying disease may lead to symptomatic improvement of C1INH-AAE.
